# Comparative Evaluation of Four Different Anti-CCP Assays for the Diagnosis of Rheumatoid Arthritis: A Diagnostic Performance Analysis

**DOI:** 10.3390/diagnostics15101293

**Published:** 2025-05-21

**Authors:** Lydia Lamara Mahammed, Tamazouzt Hadjout, Asma Bensaci, Ryma Hamma, Ghalya Bousbia, Nawel Dahmani, Halima Ismail, Nadia Tamechmacht, Reda Djidjik

**Affiliations:** 1 Department of Medical Immunology, Beni-Messous Teaching Hospital, Algiers 16000, Algeria; lyla.hadjout@gmail.com (T.H.); r.djidjik@univ-alger.dz (R.D.); 2 Faculty of Pharmacy, University of Algiers, Algiers 16000, Algeria

**Keywords:** anti-CCP antibodies, autoimmune diseases, chemiluminescence, ELISA, immunoassay, rheumatoid arthritis

## Abstract

**Background/Objectives**: Anti-cyclic citrullinated peptide (anti-CCP) antibodies are highly specific markers for rheumatoid arthritis (RA). Over the past decade, novel automating detection systems have been developed for anti-CCP detection. The present study aimed to evaluate the diagnostic performances of three fully automated anti-CCP assays in comparison to a conventional manual enzyme-linked immunosorbent assay (ELISA). **Methods**: One hundred ninety-nine patients with rheumatic symptoms (100 with RA and 99 without RA) were tested for anti-CCP autoantibodies using four assays: a manual-ELISA (EUROIMMUN^®^), two chemiluminescence immunoassays (CLIAs) performed on the MAGLUMI X3^®^ and iFlash 1800^®^ platforms, and an enzyme immunoassay (EIA) run on the UNI^®^ analyzer. **Results**: The Kappa statistic indicated a moderate qualitative agreement among the EUROIMMUN, iFlash, and UNI assays (0.734 ≤ ĸ ≤ 0.778), while the MAGLUMI anti-CCP assay showed only weak-to-moderate agreement with the others (0.510 ≤ ĸ ≤ 0.628). A strong positive correlation was observed between anti-CCP levels measured by the four assays (0.747 ≤ rho ≤ 0.839). At the manufacturers’ cut-off values, sensitivities ranged from 76% to 99% and specificities from 69.7% to 99%, depending on the assay. However, at a fixed specificity of 95%, all the four assays showed good diagnostic performances for RA, with sensitivities ranging from 80% to 89% and positive likelihood ratios (LRs+) from 16 to 17.8. **Conclusions**: Our results revealed that at the manufacturers’ cut-offs, the UNI anti-CCP assay was the most valuable alternative to the conventional ELISA for diagnosing RA in our cohort. Nevertheless, after an appropriate adjustment of the thresholds, all the evaluated assays showed good diagnostic performances for RA.

## 1. Introduction

Rheumatoid arthritis (RA) is a systemic inflammatory autoimmune disease that affects about 0.5 to 1% of the world’s population [1]. Several serological markers have been evaluated to aid in the diagnosis of RA. The most relevant ones are rheumatoid factor (RF) and anti-citrullinated protein antibodies (ACPAs), which were included in the 2010 American College of Rheumatology/European League Against Rheumatism classification criteria for RA (2010 ACR/EULAR criteria), along with clinical and radiological evaluation [2]. Although RF and ACPAs are considered equivalent in these criteria, ACPAs are more specific for RA than RF while offering similar levels of sensitivity [3].

ACPAs are a heterogeneous group of antibodies defined by their capacities to recognize post-translationally modified citrullinated proteins/peptides [4]. In clinical practice, these antibodies are mostly detected using the anti-cyclic citrullinated peptide (anti-CCP) assay. Three generations of anti-CCP assays were developed for serological diagnosis of RA: anti-CCP1 relied on a cyclic citrullinated peptide derived from the filaggrin protein as antigen, anti-CCP2 uses synthetic cyclic citrullinated peptides, and anti-CCP3, which contains multiple citrullinated epitopes, is designed in a conformational structure to enhance epitope exposure and immunoreactivity [5,6]. Due to their limited sensitivity, anti-CCP1 assays have been replaced by subsequent generations of tests to improve the diagnostic accuracy for RA [5,6]. Several studies demonstrated that anti-CCP2 and anti-CCP3 offer comparable performance characteristics in RA patients [7,8,9,10,11], whereas few reports showed that anti-CCP3 tests have better performance than anti-CCP2 assays [12,13].

Over the past years, several commercial immunoassays have been introduced for the detection of anti-CCP antibodies. Traditionally, enzyme-linked immunosorbent assays (ELISA) have been widely used because of their low cost and relatively high sensitivity and specificity [14]. Nevertheless, the manual procedure was labor and time-consuming, making it unsuitable for speedy detection. Accordingly, novel automating detection systems, such as chemiluminescent immunoassay (CLIA), electrochemiluminescence immunoassays (ECLIA), fluoroenzymatic immunoassay (FEIA), etc., have been developed for anti-CCP detection, and promising results have been yielded [10,11,12,13,15,16,17,18].

Automation makes these immunoassays very attractive, especially for high-throughput laboratories. Nevertheless, their heterogeneity regarding their antigenic composition and the choice of cut-off levels results in highly variable diagnostic performances in terms of sensitivity and specificity [10,11,12,13,15,16,17,18]. This variability highlights the critical need for rigorous evaluation of new assays prior to their use in clinical diagnosis.

In this context, the present study aimed to evaluate the diagnostic performance of three fully automated anti-CCP assays, which have been newly implemented in our laboratory, in comparison to a conventional anti-CCP assay using a manual ELISA methodology.

## 2. Materials and Methods

### 2.1. Patients

Over a course of 3 months, a total of 199 patients with rheumatic symptoms referred to the Department of Medical Immunology at Beni-Messous Teaching Hospital (Algiers, Algeria) for anti-CCP and RF testing as part of the diagnostic investigation were subsequently enrolled in the study.

Next, the subjects’ medical records were retrospectively evaluated for RA, resulting in the individualization of two groups:

- The first group of 100 patients (85 women and 15 men, mean age 51.3 ± 14.2 years, range 19–88) with a diagnosis of RA according to the 2010 ACR/EULAR classification criteria [2].
- The second group of 99 patients (80 women and 19 men, mean age 40.8 ± 18.5 years, range 4–80), for whom anti-CCP testing was requested by a physician, was ultimately diagnosed as not having RA after further workups (the control group).

The serum samples used for this study were from the serum data bank. Samples were initially obtained from patients as part of routine screening for autoantibodies in the clinical laboratory. There was no informed consent for this study, but the study was approved by the hospital’s institutional ethics committee.

### 2.2. Anti-CCP Assays

Patients’ sera were initially tested for the presence of anti-CCP antibodies by the conventional manual EUROIMMUN-anti-CCP ELISA (Euroimmun, Medizinische Labordiagnostika AG, Lübeck, Germany), which is currently used routinely in our laboratory. We subsequently stored the residual sera at −20 °C, which were thawed later for further anti-CCP measurements.

Anti-CCP antibodies were then assessed by three commercial anti-CCP assays conducted on three fully automated platforms, including two systems with chemiluminescence detection, MAGLUMI X3^®^ (Snibe Co., Ltd., Shenzhen, China) and iFlash 1800^®^ (YHLO Biotech Co., Ltd., Shenzhen, China), and an enzyme immunoassay (EIA) that is run on the UNI^®^ analyzer (NeoMedica d.o.o., Niš, Serbia). A comparison of the characteristics of these assays is summarized in Table 1.

All tests were performed according to the manufacturers’ instructions. The antibody isotype detected was IgG. The discriminating cutoffs were those recommended in the instructions for use. Note that for UNI, borderline results were considered negative.

### 2.3. Statistical Analysis

SPSS software (IBM Statistic 20.0) was used for statistical analysis and data visualization. Qualitative agreement was calculated using Cohen’s kappa (κ) with 95% confidence intervals (CIs), interpreted as follows: ≤0.20, none; 0.21–0.39, minimal; 0.40–0.59, weak; 0.60–0.79, moderate; 0.80–0.90, strong; and >0.90, nearly perfect [19]. Correlation coefficient between different assay results was determined by the Spearman rank test, which was assessed as follows: ≤0.10, negligible; 0.11–0.39, weak; 0.40–0.69, moderate; 0.70–0.89, strong; and >0.90, very strong [20].

To evaluate the diagnostic performances of the anti-CCP assays for the diagnosis of RA, the sensitivity, specificity, and likelihood ratio (LR) of each assay were calculated using the manufacturers’ cut-off values, a cut-off value set at a level of 95% specificity, and the optimal cut-off value obtained from the receiver-operating characteristic (ROC) curve analysis. Optimal cut-off values for the four assays were calculated using the maximum Youden Index (=sensitivity + specificity − 1) to optimize combined sensitivity and specificity. *p* values less than 0.05 were considered statistically significant for all analyses.

## 3. Results

### 3.1. Prevalence of Anti-CCP Antibodies According to Each Assay

**Figure 1:**
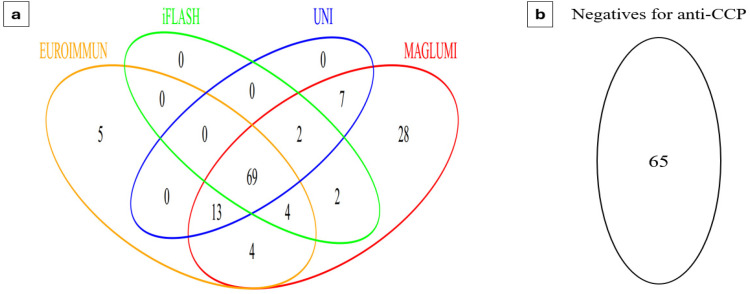
Overview of the assay results for the study population. (**a**) Patients with positive anti-CCP results in at least one of the assays. (**b**) Patients negative for anti-CCP. CCP, cyclic citrullinated peptide.

An overview of the assay results for the study population is illustrated in Figure 1. Out of the 199 patients, 134 (67.3%) tested positive for at least one of the anti-CCP assays, while 65 (32.7%) were negative regardless of the method used. Among the four evaluated assays, the MAGLUMI anti-CCP assay demonstrated the highest positivity rate, both in patients with RA and those without RA (99% and 30.3%, respectively). On the other hand, the iFlash assay had the lowest rate of positivity (76% and 1.01%, respectively) (Table 2).

### 3.2. Qualitative and Quantitative Agreement Between the Results of the Four Immunoassays

The Kappa statistic indicated a moderate qualitative agreement between the assays from EUROIMMUN, iFlash, and UNI (0.734 ≤ ĸ ≤ 0.778), while the MAGLUMI anti-CCP assay showed only weak-to-moderate agreement with the other assays (0.510 ≤ ĸ ≤ 0.628) (Table 3). Discrepant results among the four immunoassays are detailed in Table S1.

**Figure 2:**
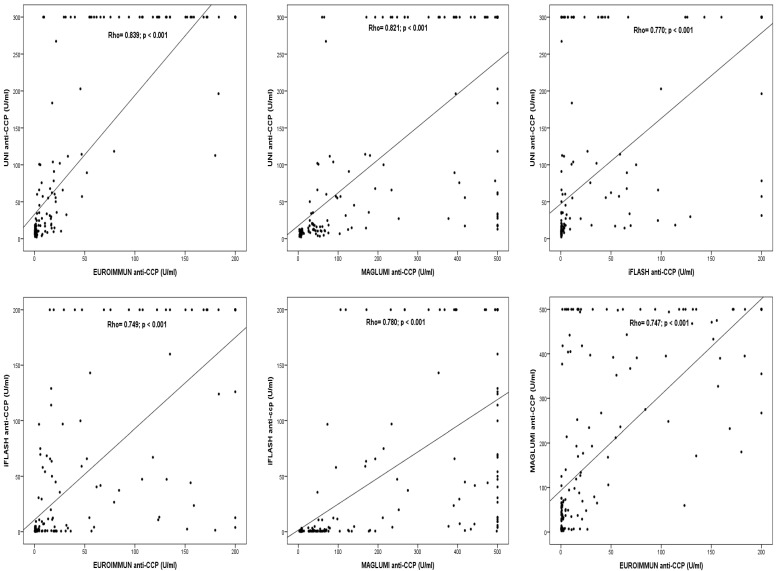
Correlations between levels of anti-CCP antibodies measured by the four evaluated assays.

Quantitative agreements between anti-CCP values were evaluated using the Spearman rank test and demonstrated strong positive significant correlations, with Spearman rho values ranging from 0.747 to 0.839 (Figure 2).

Based on the manufacturers’ cutoff values, the UNI anti-CCP assay presented the highest qualitative and quantitative agreement with the EUROIMMUN anti-CCP assay (ĸ = 0.778, total concordance rate = 89.4%, and Rho = 0.839).

### 3.3. Performance of the Four Immunoassays for Diagnosis of Patients with RA in Our Cohort

**Figure 3:**
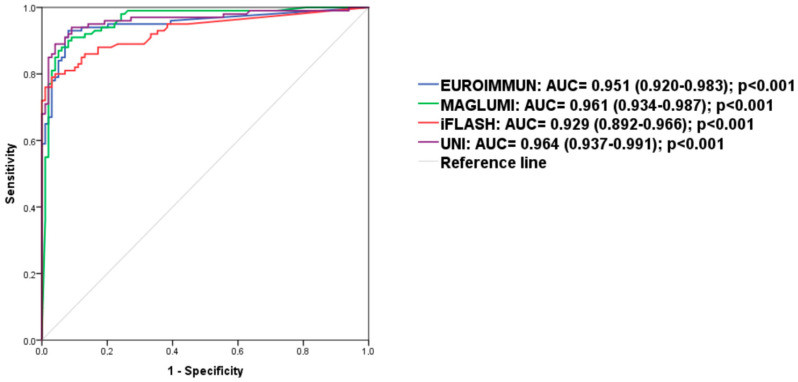
ROC curves analysis.

To compare the diagnostic performance of the tests, ROC analysis was performed (Figure 3). All anti-CCP assays showed excellent performance in discriminating between RA and non-RA (AUC > 0.9 for each test system, ranging from 0.929 to 0.964).

At the manufacturer’s cut-off values (Table 4), sensitivities ranged from 76% to 99%, and specificities from 69.7% to 99%, depending on the assay. The iFlash, UNI, and EUROIMMUN anti-CCP assays all showed positive likelihood ratios (LR+) exceeding 10, varying from 12.39 to 76, while the LR+ for the MAGLUMI anti-CCP assay was 3.27.

As strong positive results have more clinical weight for the diagnosis according to the 2010 ACR/EULAR classification criteria, we assessed the clinical performance at three times the manufacturer’s cut-off. At this threshold, the specificity reached 100% for the iFlash and UNI anti-CCP assays, 97% for EUROIMMUN, and only 86.9% for MAGLUMI, which had an LR+ of 6.9.

At the optimal cut-offs determined by the Youden index, assay sensitivities ranged from 79% to 93%, and specificities ranged from 91.9% to 97%. The optimal cut-offs for EUROIMMUN and iFlash anti-CCP assays differed slightly from those of the manufacturers’ (4 U/mL vs. 5 U/mL, respectively, for both). The adjusted cut-off for the MAGLUMI anti-CCP assay was 4.2 times higher than that of the manufacturer’s, achieving a specificity of 95% with a sensitivity of 87%. For the iFlash anti-CCP assay, an optimal cut-off of 19.9 U/mL improved the sensitivity from 87% to 89% without any loss of specificity (96%).

When the assays were compared at the same specificity (95%), the differences in sensitivity (80% to 89%) and LR+ (16 to 17.8) between them became less pronounced. The UNI anti-CCP assay provided slightly better diagnostic performance than the others (sensitivity 89% and LR+ 17.8).

## 4. Discussion

ACPA antibodies are key serological markers for the diagnosis of RA. According to the 2010 ACR/EULAR classification criteria [2], the presence of ACPA at low levels contributes two points, while high levels, >3 times the manufacturer’s cut-off value, contribute three points. Therefore, ACPA can account for up to 50% of the score required to classify RA, highlighting its major importance in the 2010 classification criteria. Consequently, several tests have been developed for the detection of ACPA using the anti-cyclic citrullinated peptide (anti-CCP) assay. Although ELISA has been used for many years and remains in practice, the introduction of fully automated instruments in recent years has led to rapid adoption in autoimmune disease diagnostics. However, despite the availability of reference preparation from the Antibody Standardization Committee (ASC), a subcommittee of the International Union of Immunological Societies (IUIS) quality-assessment and standardization committee [21], to the best of our knowledge, no commercial ACPA test has been standardized against this preparation to date [22]. In the absence of such standardization, a rigorous evaluation of new assays prior to their use in clinical diagnosis remains essential.

Among the new methodologies used, chemiluminescence immunoassay (CLIA) has gained widespread adoption in clinical disease diagnosis, notably for autoantibody detection, owing to its rapid detection speed and ease of operation [23]. Numerous CLIA-based anti-CCP assays have shown promising results [10,11,12,15,16,18]. In our study, two commercial anti-CCP CLIA assays conducted on two fully automated platforms, MAGLUMI X3^®^ and iFlash 1800^®^, were evaluated and compared to the conventional EUROIMMUN anti-CCP ELISA. To the best of our knowledge, no prior study has assessed the diagnostic performance of the MAGLUMI anti-CCP assay, while the iFlash anti-CCP assay has already been evaluated [18]. The third analyzer, UNI^®^, which uses an immuno-enzymatic detection method, is also being evaluated for the first time.

To compare the diagnostic performance of these tests, ROC analysis was performed. In this comparison, patients who initially underwent anti-CCP antibody testing but were later found not to have RA were considered the control group. All four anti-CCP assays showed excellent performance in discriminating between RA and non-RA patients (AUC > 0.9), suggesting there is no advantage in using one test over another. However, focusing solely on AUC when comparing assays can be misleading, as this metric represents an overall measure of accuracy and includes clinically relevant and irrelevant thresholds in a single value [24]. Therefore, diagnostic accuracy should be presented at specific thresholds by using paired measures such as sensitivity and specificity, or by combining the two parameters into one measure called the likelihood ratio (LR) [25]. At the manufacturers’ cut-off values, sensitivities ranged from 76% to 99%, and specificities from 69.7% to 99%, depending on the assay, with widely varying LRs+ values (3.27 to 76). Moreover, the rates of total concordance and qualitative agreement between assays were also highly variable.

The discrepancies observed between the four assays may be related to several factors. First, differences in the type and source of the antigens. Although all the evaluated assays use second-generation CCP, differences in the nature and structure of the peptides could significantly influence assay performance [26,27]. This is particularly relevant since ACPA represents a heterogeneous group of antibodies with different subtypes and affinities [4,28]. Unfortunately, the sequences and immunological characteristics of the citrullinated antigens were not available to us since they are proprietary sequences owned by the companies. Second, technical differences among the assays, including variations in methods of quantification and cut-off values, may have contributed to the observed discrepancies. The choice of cut-off values plays a crucial role in determining diagnostic performance. While manufacturer-recommended thresholds are widely used in clinical practice, they may not always be optimal for every assay.

In our study, at the manufacturers’ designated cut-off values, the MAGLUMI anti-CCP assay demonstrated the lowest specificity (69.7%), with an LR+ of 3.27 indicating limited ability to confirm RA diagnosis and a higher risk of false positive results. Consequently, this could lead to overdiagnosis and potentially unnecessary treatment in patients who do not have RA. Notably, one-third of the non-RA patients in our cohort tested positive for anti-CCP antibodies. In such a case, cut-off adjustments may be necessary to improve diagnostic accuracy and achieve a more clinically relevant specificity. Therefore, we evaluated the optimal anti-CCP cut-off values for each test using ROC curve analysis, applying the Youden index to optimize combined sensitivity and specificity. We observed that the adjusted thresholds were very slightly different from those of the manufacturer’s values for EUROIMMUN, iFlash, and UNI, while it was four times higher for MAGLUMI. This substantial increase in the MAGLUMI threshold resulted in a marked improvement in specificity, rising from 69.7% to 95%, which is crucial for distinguishing RA from other clinically similar conditions and reducing the risk of overdiagnosis and overtreatment. These findings underscore the importance of tailoring cut-off values to the studied population to optimize diagnostic accuracy [12].

Finally, to allow a more standardized comparison of the diagnostic performance across the four assays, we adjusted the clinical specificity to 95%, ensuring that all tests were evaluated under the same conditions. At this specificity, the differences in sensitivities and LR+ values between the four assays became less pronounced. Similar observations have been reported with other anti-CCP assays when the cut-offs were adjusted to the same diagnostic specificity [16]. As a result, some authors have suggested that harmonizing ACPA results, by adopting a common high-specificity threshold, could help standardize interpretation across different assays and aid clinical decision-making [22]. This approach could reduce inter-assay variability and assist clinicians in more accurately interpreting the clinical significance of laboratory findings. In line with this, our results showed that when specificity was aligned at 95%, all four assays maintained good diagnostic performances for RA, with sensitivities ranging from 80% to 89% and LRs+ ranging from 16 to 17.8. Nevertheless, the UNI anti-CCP assay demonstrated slightly better diagnostic performances than the others (the highest sensitivity and LR+). Considering these results, along with the high qualitative agreement between UNI and EUROIMMUN, UNI seems to be the most reliable alternative to the manual-ELISA to diagnose RA in our cohort.

This study has several limitations that should be noted. Firstly, the sample size was relatively small, and it was conducted at a single center, which may restrict the generalizability of the findings. Nevertheless, it is important to highlight that fully automated methods for autoimmune disease diagnosis have only recently been introduced into routine clinical practice in Algeria. Many laboratories have yet to implement these automated platforms and remain hesitant to transition from manual ELISA to fully automated methods. In this context, our study represents a critical first step in evaluating the feasibility and diagnostic performance of these technologies in a local setting. While these initial findings provide valuable insights, further validation through larger, multi-center studies is essential to strengthen our findings. Secondly, we did not evaluate the analytical performances of the four assays, which may impact the comparability of the results. Thirdly, we did not include a healthy population as controls. Nevertheless, disease controls consisting of patients with a clinical phenotype mimicking the target diagnosis, as in our study, are better comparators for defining clinically significant levels in the real-world clinical practice.

## 5. Conclusions

Our results revealed that at the manufacturers’ cut-offs, the UNI anti-CCP assay was the most valuable alternative to the conventional ELISA to diagnose RA in our cohort. Nevertheless, when evaluated at the same specificity of 95%, all four assays showed good diagnostic performances for RA, with sensitivities ranging from 80% to 89% and LRs+ ranging from 16 to 17.8. Therefore, clinical laboratories need to establish their own cut-off values for each newly implanted assay, depending on the local population for RA diagnosis. This is illustrated in our study with the MAGLUMI anti-CCP assay, where an appropriate adjustment of the threshold (>4 times the manufacturers’ cut-off) notably improved the specificity from 69.7% to 95%.

## Figures and Tables

**Table 1:** Characteristics of anti-CCP assays.

	EUROIMMUN	MAGLUMI X3	iFlash 1800	UNI
Manufacturer	EUROIMMUN	Snibe	YHLO	NeoMedica
Principal of assay	ELISA	CLIA	CLIA	EIA
Citrullinated antigen	Second-generation synthetic CCPs	Second-generation synthetic CCPs	Second-generation synthetic CCPs	Second-generation synthetic CCPs
Range detection (U/mL)	0.3–200	0.783–500	0.5–200	0.5–300
Conjugate	Peroxidase-labelled anti-human IgG	ABEI labeled anti-human IgG	Acridinium-ester-labeled anti-human IgG	HRP-labelled anti-human IgG
Interpretation of the results (U/mL)	≤5 negative >5 positive	<17 negative ≥17 positive	>5 negative ≥5 positive	>18 negative 18–22 borderline >22 positive

ABEA, aminobutylethylisoluminol; CCP, cyclic citrullinated peptide antibody; CLIA, Chemiluminescence immunoassay; EIA, enzyme immunoassay; ELISA, enzyme-linked immunosorbent assay; HRP, horseradish peroxidase; IgG, immunoglobulin G.

**Table 2:** Prevalence of anti-CCP antibodies according to disease groups.

Diagnostic Group	Number (%) of Positive Results			
	EUROIMMUN	MAGLUMI	iFlash	UNI
**RA (*N* = 100)**	88 (88%)	99 (99%)	76 (76%)	87 (87%)
**Non-RA (*N* = 99)**	7 (07.07%)	30 (30.30%)	1 (01.01%)	4 (04.04%)

CCP, cyclic citrullinated peptide antibody; RA, rheumatoid arthritis.

**Table 3:** Qualitative agreement between anti-CCP assays at manufacturer’s cutoff.

	EUROIMMUN	MAGLUMI	iFlash
UNI Concordance rate; n (%) Agreement	178 (89.4%) ĸ = 0.778 *p* > 0.001 95% CI [0.690–0.866]	161 (80.9%) ĸ = 0.628 *p* > 0.001 95% CI [0.530–0.726]	173 (86.9%) ĸ = 0.734 *p* > 0.001 95% CI [0.640–0.828]
iFlash Concordance rate; n (%) Agreement	173 (86.9%) ĸ = 0.736 *p* > 0.001 95% CI [0.644–0.828]	147 (70.4%) ĸ = 0.510 *p* > 0.001 95% CI [0.410–0.610]	
MAGLUMI Concordance rate; n (%) Agreement	155 (77.9%) ĸ = 0.564 *p* > 0.001 95% CI [0.456–0.672]		

**Table 4:** Diagnostic performance characteristics of anti-CCP assays.

	EUROIMMUN	MAGLUMI	iFlash	UNI
Manufacturer’s cut-off (U/mL) Sensitivity (95% CI) Specificity (95% CI) LR+ LR−	5 88 (83.4–92.5) 92.9 (89.3–96.5) 12.39 0.13	17 99 (97.6–99.9) 69.7 (63.3–76.1) 3.27 0.01	5 76 (70.1–81.9) 99 (97.6–99.9) 76 0.24	22 87 (82.3–91.7) 96 (93.3–98.7) 21.75 0.14
3× Manufacturer’s cut-off (U/mL) Sensitivity (95% CI) Specificity (95% CI) LR+ LR−	15 76 (70.1–81.9) 97 (94.6–99.4) 25.3 0.25	51 91 (87–95) 86.9 (82.2–91.6) 6.9 0.10	15 64 (57.3–70.7) 100 / 0.36	66 68 (61.5–74.5) 100 / 0.32
Optimal cut-off (U/mL) Sensitivity (95% CI) Specificity (95% CI) LR+ LR−	4 93 (89.5–96.5) 91.9 (88.1–95.7) 11.5 0.07	71.7 87 (82.3–91.7) 95 (92–98) 17.4 0.14	4 79 (73.3–84.7) 97 (94.6–99.4) 26.3 0.21	19.9 89 (84.7–93.4) 96 (93.3–98.7) 22.3 0.11
Cut-off at 95% specificity (U/mL) Sensitivity (95% CI) LR+ LR−	8 84 (78.9–89.1) 16.8 0.17	71.7 87 (82.3–91.7) 17.4 0.14	3.4 80 (74.4–85.6) 16 0.21	19.2 89 (84.7–93.4) 17.8 0.12

CCP, cyclic citrullinated peptide; LR, likelihood ratio.

## Data Availability

The raw data supporting the conclusions of this article will be made available by the authors on request.

## Supplementary Materials

The following supporting information can be downloaded at: https://www.mdpi.com/article/10.3390/diagnostics15101293/s1. Table S1: Discrepant results among the four anti-CCP assays (*n* = 65).

## References

[1] Finckh A., Gilbert B., Hodkinson B., Bae S.-C., Thomas R., Deane K.D., Alpizar-Rodriguez D., Lauper K. (2022). Global epidemiology of rheumatoid arthritis. Nat. Rev. Rheumatol..

[2] Aletaha D., Neogi T., Silman A.J., Funovits J., Felson D.T., Bingham C.O., Birnbaum N.S., Burmester G.R., Bykerk V.P., Cohen M.D. (2010). 2010 Rheumatoid arthritis classification criteria: An American College of Rheumatology/European League Against Rheumatism collaborative initiative. Arthritis Rheum..

[3] Nishimura K., Sugiyama D., Kogata Y., Tsuji G., Nakazawa T., Kawano S., Saigo K., Morinobu A., Koshiba M., Kuntz K.M. (2007). Meta-analysis: Diagnostic accuracy of anti-cyclic citrullinated peptide antibody and rheumatoid factor for rheumatoid arthritis. Ann. Intern. Med..

[4] Liu J., Gao J., Wu Z., Mi L., Li N., Wang Y., Peng X., Xu K., Wu F., Zhang L. (2022). Anti-citrullinated Protein Antibody Generation, Pathogenesis, Clinical Application, and Prospects. Front. Med..

[5] Alghamdi M.F., Redwan E.M. (2021). Advances in the diagnosis of autoimmune diseases based on citrullinated peptides/proteins. Expert. Rev. Mol. Diagn..

[6] Rahali F.Z., Tarmidi M., Hazime R., Admou B. (2023). Clinical significance of anti-cyclic citrullinated peptide (anti-CCP) antibodies in rheumatoid arthritis: Literature review. SN Compr. Clin. Med..

[7] Lutteri L., Malaise M., Chapelle J.P. (2007). Comparison of second- and third-generation anti-cyclic citrullinated peptide antibodies assays for detecting rheumatoid arthritis. Clin. Chim. Acta Int. J. Clin. Chem..

[8] Correia M.L., Carvalho S., Fortuna J., Pereira M.H. (2008). Comparison of three anti-CCP antibody tests and rheumatoid factor in RA and control patients. Clin. Rev. Allergy Immunol..

[9] Shidara K., Inoue E., Tanaka E., Hoshi D., Seto Y., Nakajima A., Momohara S., Taniguchi A., Yamanaka H. (2011). Comparison of the second and third generation anti-cyclic citrullinated peptide antibody assays in the diagnosis of Japanese patients with rheumatoid arthritis. Rheumatol. Int..

[10] Ji M., Hur M., Moon H.W., Park M., Yun Y.M., Lee S.H. (2018). Comparison of second- and third-generation immunoassays for detection of anti-cyclic citrullinated peptide antibodies. Scand. J. Clin. Lab. Investig..

[11] Cho J., Pyo J.Y., Fadriquela A., Uh Y., Lee J.H. (2021). Comparison of the analytical and clinical performances of four anti-cyclic citrullinated peptide antibody assays for diagnosing rheumatoid arthritis. Clin. Rheumatol..

[12] Debaugnies F., Servais G., Badot V., Noubouossie D., Willems D., Corazza F. (2013). Anti-cyclic citrullinated peptide antibodies: A comparison of different assays for the diagnosis of rheumatoid arthritis. Scand. J. Rheumatol..

[13] Malher M., Bentow C., Albesa R., Cesana L., Martinez-Prat L., Roux-Lombard P., Nissen M., Lamacchia C., Gabay C. (2018). P024 Comparison of CCP2 and CCP3 assays in a large cohort of established rheumatoid arthritis and controls. Ann. Rheum. Dis..

[14] Bizzaro N., Tampoia M. (2008). Diagnostic Accuracy of Immunoassays for the Detection of Antibodies to Citrullinated Proteins. Clin. Rev. Allergy Immunol..

[15] Tanaka R., Takemura M., Sato M., Yamada Y., Nakagawa T., Horibe T., Hoshi M., Otaki H., Ito H., Seishima M. (2010). Comparison of chemiluminescence enzyme immunoassay (CLEIA) with ELISA for the determination of anti-cyclic citrullinated peptide antibodies. Clin. Chim. Acta Int. J. Clin. Chem..

[16] Webb T., Lakos G., Swart A., Gürtler I., Favalli E.G., Schioppo T., Mahler M. (2014). Clinical evaluation of a novel chemiluminescent immunoassay for the detection of anti-citrullinated peptide antibodies. Clin. Chim. Acta Int. J. Clin. Chem..

[17] Vos I., Van Mol C., Trouw L.A., Mahler M., Bakker J.A., Van Offel J., De Clerck L., Huizinga T.W. (2017). Anti-citrullinated protein antibodies in the diagnosis of rheumatoid arthritis (RA): Diagnostic performance of automated anti-CCP-2 and anti-CCP-3 antibodies assays. Clin. Rheumatol..

[18] Ma L., Wang W., Li L., Chen Y., Chen B., Shao M., Cheng Y., Zhou R. (2022). Comparison of different assays for the detection of anticyclic citrullinated peptide antibodies in patients with rheumatoid arthritis. Front. Immunol..

[19] McHugh M.L. (2012). Interrater reliability: The kappa statistic. Biochem. Medica.

[20] Schober P., Boer C., Schwarte L.A. (2018). Correlation Coefficients: Appropriate Use and Interpretation. Anesth. Analg..

[21] International Union of Immunological Societies (IUIS)/Antibody Standardization Committee (ACS) Reference Reagent for Human Reference Serum for Citrullinated Peptide/Protein Antibodies (ACPA). https://asc.dental.ufl.edu/reference-sera/.

[22] Rönnelid J., Turesson C., Kastbom A. (2021). Autoantibodies in Rheumatoid Arthritis—Laboratory and Clinical Perspectives. Front. Immunol..

[23] Cinquanta L., Fontana D.E., Bizzaro N. (2017). Chemiluminescent immunoassay technology: What does it change in autoantibody detection?. Auto-Immun. Highlights.

[24] Eusebi P. (2013). Diagnostic accuracy measures. Cerebrovasc. Dis..

[25] Akobeng A.K. (2007). Understanding diagnostic tests 1: Sensitivity, specificity and predictive values. Acta Paediatr..

[26] Schellekens G.A., de Jong B.A., van den Hoogen F.H., van de Putte L.B., van Venrooij W.J. (1998). Citrulline is an essential constituent of antigenic determinants recognized by rheumatoid arthritis-specific autoantibodies. J. Clin. Investig..

[27] Ru Z., Zhang H., Huang X., Lou J., Liao J., Chen Z., Yang X. (2022). A new pattern of citrullinated peptides improves the sensitivity for diagnosing rheumatoid arthritis. Clin. Biochem..

[28] Catrina A., Krishnamurthy A., Rethi B. (2021). Current view on the pathogenic role of anti-citrullinated protein antibodies in rheumatoid arthritis. RMD Open.

