# Supplementary material for: Comparative Evaluation of Four Different Anti-CCP Assays for the Diagnosis of Rheumatoid Arthritis: A Diagnostic Performance Analysis
Source: Diagnostics (Basel). 2025 May 21;15(10):1293. doi: 10.3390/diagnostics15101293 (PMC12110532; doi:10.3390/diagnostics15101293)
Supplement: Supplementary file 1 [file diagnostics-15-01293-s001.zip › diagnostics-3546335-supplementary.pdf]

Table S1. Discrepant results among the four anti-CCP assays (n=65).

**Profile of patients**		**Diagnosis**	**RF**	**Results of anti-CCP antibodies**				**Interpretation of discrepant results***
				**EUROIMMUN**	**MAGLUMI**	**iFlash**	**UNI**	
**Profile 1**	n=23	Non-RA	Neg	Neg	**Pos**	Neg	Neg	Probably false Positive (MAGLUMI)
	n=5	RA	Pos					
**Profile 2**	n=4	Non-RA	Neg	**Pos**	Neg	Neg	Neg	Probably false Positive (EUROIMMUN)
	n=1	Non-RA	Pos					
**Profile 3**	n=4	Non-RA	Pos/Neg	Neg	**Pos**	Neg	**Pos**	Indeterminate
	n=3	RA						
**Profile 4**	n=2	Non-RA	Neg	**Pos**	**Pos**	Neg	Neg	Indeterminate
	n=2	RA	Pos					
**Profile 5**	n=1	Non-RA	Neg	Neg	**Pos**	**Pos**	Neg	Indeterminate
	n=1	RA	Pos					
**Profile 6**	n=13	RA	Pos/Neg	**Pos**	**Pos**	Neg	**Pos**	Probably false Negative (iFLASH)
**Profile 7**	n=4	RA	Pos/Neg	**Pos**	**Pos**	**Pos**	Neg	Probably false Negative (UNI)
**Profile 8**	n=2	RA	Pos	Neg	**Pos**	**Pos**	**Pos**	Probably false Negative (EUROIMMUN)

*The results were considered true in the case of same results in three assays among the four assays. Positive results were presented as bold letterings.

CCP, cyclic citrullinated peptide. NEG: negative; POS: positive; RA: rheumatoid arthritis; RF: rheumatoid factor.
